# Characterization of *SR3 *reveals abundance of non-LTR retrotransposons of the RTE clade in the genome of the human blood fluke, *Schistosoma mansoni*

**DOI:** 10.1186/1471-2164-6-154

**Published:** 2005-11-04

**Authors:** Thewarach Laha, Nonglack Kewgrai, Alex Loukas, Paul J Brindley

**Affiliations:** 1 Department of Parasitology, Faculty of Medicine, Khon Kaen University, Khon Kaen 40002, Thailand; 2 Division of Infectious Diseases & Immunology, Queensland Institute of Medical Research, Brisbane, Queensland, 4029, Australia; 3 Department of Tropical Medicine, and Center for Infectious Diseases, Tulane University, Health Sciences Center, New Orleans, Louisiana, 70112, USA

## Abstract

**Background:**

It is becoming apparent that perhaps as much as half of the genome of the human blood fluke *Schistosoma mansoni *is constituted of mobile genetic element-related sequences. Non-long terminal repeat (LTR) retrotransposons, related to the LINE elements of mammals, comprise much of this repetitive component of the schistosome genome. Of more than 12 recognized clades of non-LTR retrotransposons, only members of the CR1, RTE, and R2 clades have been reported from the schistosome genome.

**Results:**

Inspection of the nucleotide sequence of bacterial artificial chromosome number 49_J_14 from chromosome 1 of the genome of *Schistosoma mansoni *(GenBank AC093105) revealed the likely presence of several RTE-like retrotransposons. Among these, a new non-LTR retrotransposon designated *SR3 *was identified and is characterized here. Analysis of gene structure and phylogenetic analysis of both the reverse transcriptase and endonuclease domains of the mobile element indicated that *SR3 *represented a new family of RTE-like non-LTR retrotransposons. Remarkably, two full-length copies of *SR3*-like elements were present in BAC 49-J-14, and one of 3,211 bp in length appeared to be intact, indicating *SR3 *to be an active non-LTR retrotransposon. Both were flanked by target site duplications of 10–12 bp. Southern hybridization and bioinformatics analyses indicated the presence of numerous copies (probably >1,000) of *SR3 *interspersed throughout the genome of *S. mansoni*. Bioinformatics analyses also revealed *SR3 *to be transcribed in both larval and adult developmental stages of *S. mansoni *and to be also present in the genomes of the other major schistosome parasites of humans, *Schistosoma haematobium *and *S. japonicum*.

**Conclusion:**

Numerous copies of *SR3*, a novel non-LTR retrotransposon of the RTE clade are present in the genome of *S. mansoni*. Non-LTR retrotransposons of the RTE clade including *SR3 *appear to have been remarkably successful in colonizing, and proliferation within the schistosome genome.

## Background

Schistosomiasis is considered among the most important of the tropical diseases in terms of morbidity and mortality, ranking only behind malaria [1]. International efforts are underway to sequence the entire genomes of two of the three major schistosome species, *S. mansoni *and *S. japonicum *[2]. It is anticipated that an enhanced understanding of the schistosome genome will aid in the control of this disease, including the development of vaccines and new anti-parasite medications [3]. Up to half of the schistosome genome may be composed of repetitive sequences, including LTR and non-LTR retrotransposons, mobile genetic elements that transpose through an RNA intermediate (reviewed by Brindley et al. [4]). Mobile genetic elements are drivers of genome evolution [5,6]. In addition to this role, from a practical perspective mobile genetic elements offer potential as transgenesis vectors [7]. Problematically, however, their interspersed, repetitive nature can impede progress during genome sequencing using shotgun sequencing approaches through the confounding effects of their repetitions on sequence assembly algorithms [8,9]. For these and other reasons, we and others have been characterizing the retrotransposons of the schistosome genome [10-15]. Here we report a novel non-LTR retrotransposon termed *SR3*, a member of the RTE clade of non-LTR retrotransposons, from the genome of *S. mansoni*. Based on the multi-copy, interspersed nature of *SR3*, and the presence of other RTE elements characterized previously from the genomes of schistosomes, it appears that members of the RTE clade may be the most common and successful of the non-LTR retrotransposons to have colonized the genomes of these metazoan parasites.

## Results and Discussion

### New retrotransposons identified in bacterial artificial chromosome 49_J_14 from the genome of *S. mansoni*

**Figure 1:**
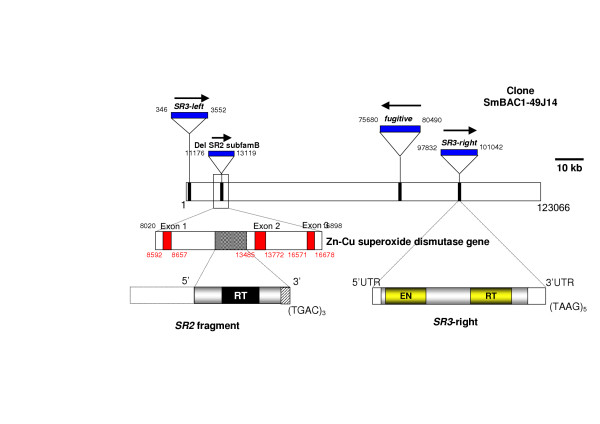
Schematic diagram of the location, size and structure of copies of the *SR3 *non-LTR retrotransposon in bacterial artificial chromosome number 49_J_14 from chromosome 1 of the genome of *Schistosoma mansoni*. The location of copies of the *SR2 *and *fugitive *retrotransposons is also presented. The arrows indicate the direction of transcription of the mobile elements. The numbers on each bar indicate the nucleotide position of the elements within the bacterial artificial chromosome. A degenerate copy of the *SR2 *element was evident within intron 1 of Zn-Cu superoxide dismutase (SOD) gene, in particular within intron 1 of the gene: three exons of the SOD are indicated in red, with the position and structure of the degenerate copy of *SR2 *indicated within intron 1. On the bottom right, a schematic of the length and domain structure of the *SR3-right *copy of the *SR3 *retrotransposon is presented. RT, reverse transcriptase; EN, endonuclease.

BLASTn searches revealed the presence of reverse transcriptase (RT)-encoding sequences in the *S. mansoni *bacterial artificial chromosome (BAC) number 49_J_14 [16], the entire sequence of which has been deposited in GenBank with accession number AC093105 by El Sayed and co-workers [3]. Annotation provided with GenBank AC093105 indicated that the sequence included in BAC 49_J_14 is from chromosome 1 of the genome of *S. mansoni*. Inspection of the nucleotide sequence of BAC 49_J_14, of ~123 kb in length, indicated the presence of a number of discrete retrotransposons. One of these encodes a novel long terminal repeat (LTR) retrotransposon, which we have described in a recent report [11] (Fig. 1). In addition, at least three non-LTR retrotransposons appeared to be located in BAC 49_J_14. One of these appeared to be a degenerate copy of an *SR2 *element. *SR2 *elements are non-LTR retrotransposons of the RTE clade [17] which are present in high copy (estimated at up to 10,000 copies) in the genome of *S. mansoni *[10,18]. This fragment of *SR2 *was located between nucleotide residues numbers 11,176 and 13,119 of BAC 49_J_14 and, more specifically appeared to be located within intron number 1 of the gene encoding cytosolic Zn/Cu superoxide dismutase [19]. As illustrated in Fig. 1, the Cu/Zn superoxide dismutase gene is present in BAC 49_J_14 between residues 8,020 and 16,898 of BAC 49_J_14. The copy of *SR2 *in the intron of the Cu/Zn superoxide dismutase gene is ~1,830 nucleotides (nt) in length, and included regions encoding the retrotransposon RT domain (Fig. 1). Over the putative RT-encoding region, the sequence was 47% identical to the RT sequence of *SR2*. At only ~1.8 kb in length, and since full-length copies of *SR2 *are ~3.9. kb in length [18], this appears to be a truncated copy of *SR2 *that is unlikely to be autonomously mobile. In like fashion to the location of this truncated copy of *SR2*, copies of other *SR2 *elements (and indeed other retrotransposons) have been identified previously in introns of other *S. mansoni *protein encoding genes [20,21].

### SR3 represents a new family of non-LTR retrotransposon from the genome of *S. mansoni*

In addition to the *fugitive *LTR retrotransposon [11], and the truncated copy of *SR2*, at least two other retrotransposons were readily identifiable in BAC 49_J_14. The first of these was located between nt 346 and 3,552 (i.e., 3,207 bp in length), and the second between nt 97,832 and 101,042 (3,211 bp in length). Comparison of the sequences of these two prospective retrotransposons revealed that they were closely related to one another and appeared to represent discrete copies of a novel family of retrotransposons. We have termed the new retrotransposon *SR3*, whose phylogenetic analysis indicated a new family of the RTE clade of non-LTR retrotransposons (see below). (SR3 stands for Schistosome Retrotransposon 3 because two other non-LTR retrotransposons described previously from *S. mansoni *are termed *SR1 *and *SR2 *[18,22]). (A recent article, published after this present report was submitted for publication, identified a *SR3*-like element in the *S. mansoni *transcriptome, termed *Perere-3*, and also identified several other novel retrotransposons [15].) For convenience of description, we refer here to the copy of *SR3 *resident between nt 346 and 3,552 of BAC 49_J_14 as *SR3-left *and the other copy between nt 97,842 and 101,042 as *SR3-right*, because they are located on the left and right sides of the BAC as in Figure 1. The full-length *SR3-left *and *SR3-right *elements were comprised of a single, read through open reading frame (ORF) encoding two functional domains similar to apurinic-apyrimidic (AP) endonuclease (EN) and RT, in that order. The element terminated with a short repeat sequence, (TAAG)_4 _or (TAAG)_5 _(Fig. 1). The nucleotide and deduced amino acid sequences of the *SR3-left *and *SR3-right *copies are provided in Additional files 1 and 2, respectively.

The sequence of 3,211 bp long *SR3-right *element translated into a single, deduced open reading frame (ORF) of 922 amino acid residues that did not include any apparent frameshift or stop codon mutations (Additional file 2). By contrast, the deduced ORF of *SR3-left *was interrupted by stop codons at amino acid positions 719 and 913 of the ORF (Additional files 1, 3). *SR3-right *has a longer terminal repeat unit than *SR3-left*, (TAAG)_5 _compared with (TAAG)_4_, which accounts for the difference in total lengths of the two copies (3,207 and 3,211 bp). (By contrast, comparison of the ORFs of *Perere-3 *(Accession CAJ00236.1) and the *SjR2 *retrotransposon (AY027869) of *S. japonicum*, with the deduced ORFs of both *SR3-left *and *SR3-right *revealed that the similarity extends well beyond the predicted ORF of 922 deduced amino acids of *SR3-right *[not shown]. Whereas this suggests the possibility of premature stop codon in the SR3 copies presented here, it may also simply reflect phylogenetic relatedness in the carboxy-terminal encoding regions and 3'UTRs of these elements.) Nonetheless, *SR3-left *and *SR3-right *are very similar to each other in sequence, with the ORFs region exhibiting 94 % identity and 97 % similarity over the predicted ORF of 922 residues (Additional file 3). Together, these findings suggest that both *SR3-left *and *SR3-right *are full-length copies and, moreover, that *SR3-right *is an intact, putatively functional and active copy, capable of autonomous retrotransposition activity. It was remarkable not only that two copies (*SR3-left *and *SR3-right*) of this retrotransposon reside in close proximity to each other in the region of the *S. mansoni *genome represented by BAC 49_J_14, but also that both copies are full-length and intact or close to intact. Most copies of non-LTR retrotransposons are 5'-truncated, due to deficits in their elongation processes, and generally include deletions or insertions (indels), and are thereby rendered inactive [6,23,24].

Four other non-LTR retrotransposons have been reported from the genome of *S. mansoni*. These are *SR1 *and *Perere*, discrete members of the CR1 clade, and *SR2 *and *Perere-3*, members of the RTE-1 clade [14,15,18,22]. *SR3 *was dissimilar to these non-LTR retrotransposons reported previously from the genome of *S. mansoni*: when compared with the deduced amino acid sequence of the ORF of *SR3*, *SR1 *shared 23 %/ 38 % amino acid sequence identity/similarity with *SR3*, *Perere *shared 22 %/35 % identity/similarity, *SR2 *shared 39 %/55 % identity/similarity and *Perere-3 *shared 78 %/88 % amino acid sequence identity/similarity with *SR3 *(not shown). Together, these differences indicated that *SR3 *was a novel element distinct from these other schistosome non-LTR retrotransposons.

### SR3 represents a new member of a family of the RTE-1 non-LTR retrotransposons

**Figure 2:**
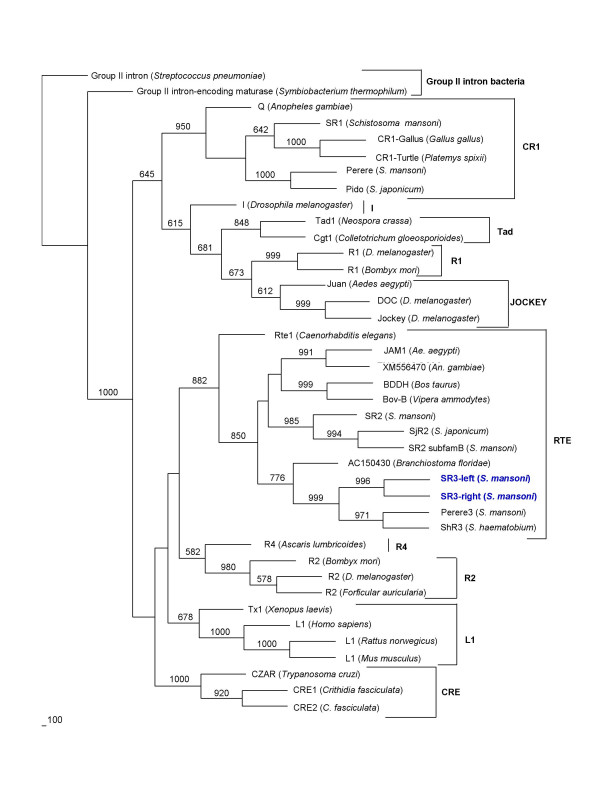
Phylogram constructed using the neighbor-joining method to compare the relationships among reverse transcriptases of *SR3 *and of representative elements belonging to the major clades of non-LTR retrotransposons [25] from a range of host genomes. Representatives of 11 clades of non-LTR retrotransposons including the RTE, CR1, L1, R1 and Jockey clades were included in the analysis. Bootstrap values, where 500 or greater from a maximum of 1,000 replicates, are presented at the nodes.

**Figure 3:**
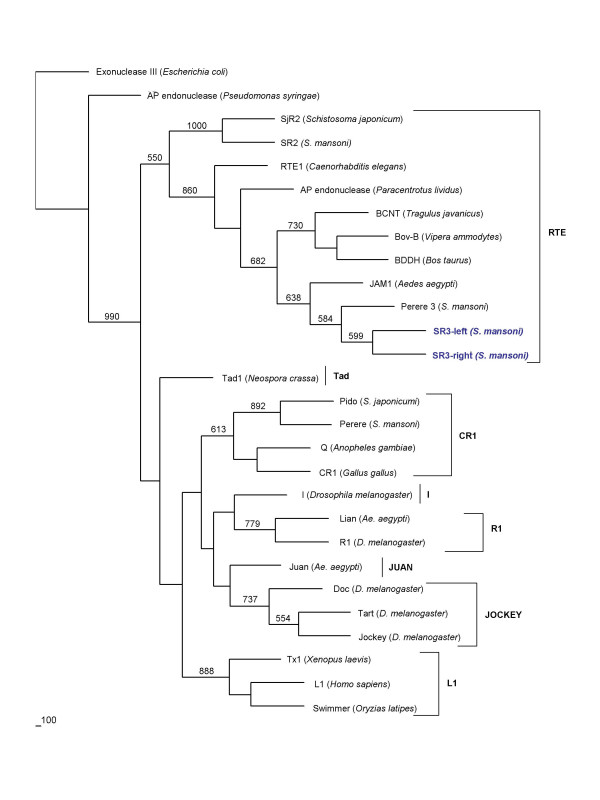
Phylogram constructed using the neighbor-joining method to compare the relationships among endonucleases of *SR3 *and of representative elements belonging to the major clades of non-LTR retrotransposons [25] from a range of host genomes. Representatives of eight major clades of non-LTR retrotransposons including the RTE, L1, CR1, Jockey, and I clades were included in the analysis. Bootstrap values of 500 or greater from 1,000 replicates are presented at the nodes.

The predicted RT domain of *SR3 *was aligned with orthologous domains of numerous other non-LTR retrotransposons including representatives from 11 clades of non-LTR retrotransposons, as defined by Eickbush and colleagues [25,26]. Phylogenetic comparison of the RT domains of these diverse elements revealed that the closest relatives of *SR3 *were *ShR3 *from *S. haematobium *and *Perere-3 *from *S. mansoni*, with close identity also to AC150430 element from *Branchiostoma floridae*, *SR2 *from *S. mansoni*, *SjR2 *from *S. japonicum *and also to *RTE-1 *from *Caenorhabditis elegans *(Figure 2; and Additional file 4), placing *SR3 *in the *RTE-1 *clade of non-LTR retrotransposons. In like fashion, a phylogenetic tree was constructed based on the EN domain of eight clades of non-LTR retrotransposons. The topography of the EN tree, and the position of *SR3 *within the RTE clade, was similar to the topography represented on the RT-based tree, confirming both the inclusion of *SR3 *as an RTE clade element and that *SR3 *and *SR2 *were discrete families of RTE-like retrotransposons (Figure 3; and Additional file 5). Indeed, in the EN tree, *SR3 *was more closely related to *RTE-1 *of *C. elegans *than to *SR2 *of *S. mansoni *(Figs. 2, 3).

### Structure of SR3

Youngman et al. [27] provided the first report of a RTE retrotransposon, from the genome of *C. elegans*. RTE clade elements display a broad host range, having been described from numerous invertebrate and vertebrate taxa, and from algae and flowering plants [14,15,17,18]. *RTE-1 *encodes a 1,066-amino-acid ORF containing both apurinic-apyrimidic endonuclease and reverse-transcriptase domains. A possible first ORF of only 43 amino acids overlaps with the larger ORF and may be the site of translation initiation. Members of the RTE clade are characterized by unusually short 3' untranslated regions that are predominantly composed of AT-rich trimer, tetramer, and/or pentamer repeats [17]. RTE-derived SINE elements are also found in mollusc and flatworm genomes.

In addition to the demonstration by phylogenetic analyses targeting both the RT and EN domains that *SR3 *is an RTE like element, we compared the structural motifs and domains of *SR3 *with *RTE-1 *of *C. elegans *and *SR2 *of *S. mansoni *in order to confirm the identity of *SR3 *as an RTE clade non-LTR retrotransposon. First, the three elements were of generally similar length; 3,291 bp for *RTE-1 *of *C. elegans *[17], 3,913 bp for *SR2 *[18], and 3,211 kb for *SR3-right*. Second, the length of the ORF was somewhat similar; 1066, 1016, and 922 amino acids for *RTE-1*, *SR2*, and *SR3 *respectively. The *RTE-1 *and *SR2 *elements may also contain a short ORF upstream of the major ORF, although this has not been confirmed by functional analysis [17,18,25]. Third, the 3'-UTRs of RTE clade elements are usually short in length and terminate in several tetrameric or pentameric, A-rich repeats. *SR3 *conformed to *RTE-1 *in this regard, with *SR3 *exhibiting a short 3'-UTR of 177 bp in length and terminating with several copies of the tetramer, TAAG (Fig. 1; Additional files 1, 2).

**Figure 4:**
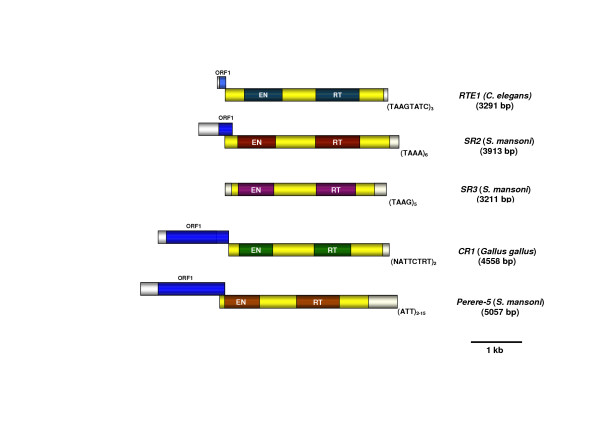
Schematic representation of the structure of non-LTR retrotransposons of the RTE and CR1 clades, including *RTE-1 *from *Caenorhabditis elegans*, *SR2 *and *SR3 *from *Schistosoma mansoni*, *CR1 *from *Gallus gallus*, and *Perere-5*, and *SR1*-like retrotransposon from *S. mansoni*. (The structure of a full-length copy of *SR1 *has not been reported [15, 22].) The sequence motifs of the 3'-termini are shown, along with positions of enzymatic domains, EN (endonuclease) and RT (reverse transcriptase). The *RTE-1 *element (3291 bp) illustrated here includes the 3'-UTR so that it is longer than the 3258 bp, described in Malik and Eickbush [17] (which included only the region between the 5'-end and the termination codon).

A schematic comparison of the structures of *RTE-1*, *SR2*, *SR3*, *CR1*, and an *SR1*-like element, *Perere-5 *[15,22], is presented in Figure 4. In summary, the *SR3 *elements of *S. mansoni *conform in all respects to the generalized structure of the RTE clade of non-LTR retrotransposons. Moreover, as with other RTE elements, SINE-like elements reported from schistosomes may be derived from *SR3*-like elements [4].

### SR3 is present in genomes of other schistosome species

Investigation of SR3 sequences in the genomes of other human schistosomes by BLAST search analysis revealed many sequences similar to *SR3 *in the transcriptomes of *S. japonicum *(e.g., GenBank AY810372, AY915175, AY813885 and AY915893). In addition, when the nucleotide sequence of *SR3-right *was employed as the query in BLASTx analysis against the GenBank non-redundant database, *SR3*-like sequences were identified within introns 1 and 6 of the gene encoding *S. haematobium *acetylcholinesterase (AChE) (GenBank AY167025) [28]. The two copies are similar in sequence (~70% identical), both copies are 5' truncated, and both include regions encoding the RT domain of the retrotransposon (not shown). The fragment within intron 1 was located between nt 1,023–2,474, and the fragment in intron 6 was located between nt 18,742–20,658. The predicted RT domain of the *SR3 *like element from *S. haematobium *(termed *ShR3*) was included in the phylogenetic tree presented in Fig. 2 and was found to be phylogenetically similar to *SR3 *from *S. mansoni*. The presence of *SR3 *elements in other schistosome species can be explained by vertical transmission from a progenitor schistosome species [29], given that vertical transmission is the expected route of transmission of non-LTR retrotransposons [24].

### Numerous copies of SR3 are interspersed throughout the genome of *S. mansoni*

**Figure 5:**
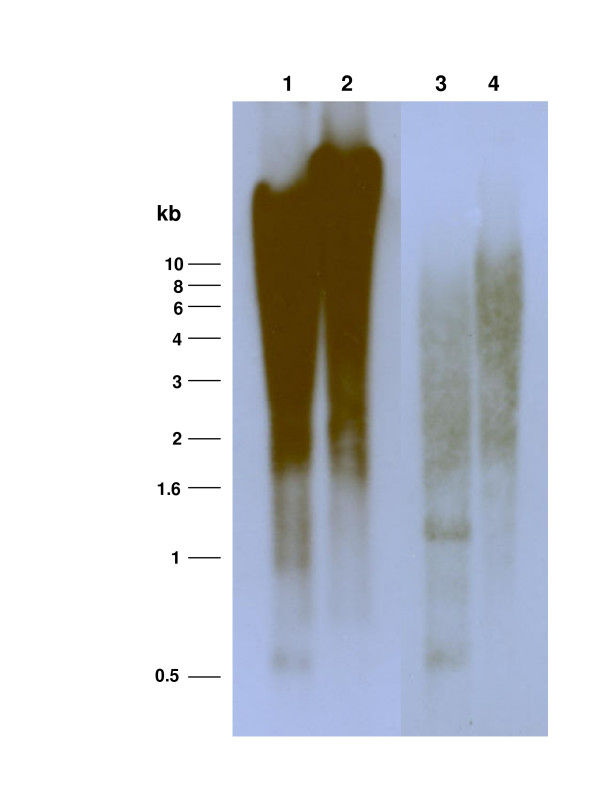
Southern hybridization analysis of genomic DNA of *S. mansoni *probed with a *SR3 *retrotransposon specific probe. Genomic DNA was cleaved with endonucleases *Hin*d III (lane 1), *Bam*H I (lane 2), *Eco*R I plus *Xba *I (lane 3) and *Hin*d III plus *Xho *I (lane 4). Molecular size standards in kilobase pairs (kb) are shown at the left. (Lanes 1 and 2 were exposed to film longer than lanes 3 and 4.)

Southern hybridization analysis revealed that multiple bands of digested genomic DNA of *S. mansoni *hybridized to the *SR3 *specific probe, indicating the presence of numerous copies of *SR3 *in the *S. mansoni *genome (Fig. 5, lanes 1 and 2). Hybridization to the gDNA fragments released by double enzyme digestions revealed an even more smeared pattern (Fig. 5, lanes 3, 4), clearly suggesting that *SR3 *elements have interspersed throughout the genome of *S. mansoni*. In addition, a bioinformatics analysis using the approach of Copeland et al. [13] was used to estimate copy number of *SR3 *by comparisons with reference copy number estimates of other mobile genetic elements and genes reported previously. BLASTn searches were undertaken using the nucleotide sequences of these reference genes and the complete nucleotide sequence of *SR3-right*. Because the construction of the *S. mansoni *BAC library (from which BAC 49_J_14 was isolated) involved partial digestion of the genomic DNA with *Hin*d III [16], genes without *Hin*d III sites will be underrepresented in the BAC end sequences. Accordingly, since sequenced BAC ends from this library constitute a large proportion of the genomic *S. mansoni *sequences in the public domain, we used only genes containing *Hin*d III sites as reference sequences. As shown in Table 1, the number of hits for SR3, 110, was higher than the number of hits for the single-copy cathepsin D gene (0 hits) and for three high copy number retrotransposons *Boudicca *(100 hits, 1,000–10,000 reported copies), *SR2 *(102 hits, 1,000–10,000 copies), and *SR1 *(104 hits, 200–2,000 reported copies) but lower than that for the multiple-copy 28S ribosomal RNA gene (157 hits) (100–200 copies). Although it is difficult with these available data to obtain a good estimate of the number of copies, however a comparison with the other 3 retrotransposons would give a tentative copy number for SR3 of between 1,000 and 10,000.

**Table 1:** Estimation by bioinformatics approaches of gene copy number of the *SR3 *non-LTR retrotransposon in the genome of *Schistosoma mansoni*.

**Gene**	**GenBank Accession**	**Query Length (bp)**	**Number of hits (Expect 0.000001)**	**Copy number**	**Key references**
Cathepsin D, Intron 4	AY309267	1636	0	1	[20]
28S rRNA	Z46503	1694	157	100–200	[46]
*Sinbad*	AY506538	6288	38	50	[13]
*Boudicca*	AY662653	5858	100	1,000–10,000	[12]
***SR3***		3211	110	>1,000	This study
*SR2*	AF025672	3913	102	1,000–10,000	[18]
*SR1*	U66331	2337	104	200–2,000	[22]
*Saci-2*	BK004069	4946	107	85–850*	[14]
*Saci-1*	BK004068	5980	133	70–700*	[14]

### SR3 is transcribed in all developmental stages of *S. mansoni*

The nucleotide sequences of the full length of *SR3-left *and *SR3-right *elements were employed as query sequences for BLAST searches of the GenBank EST database of *S. mansoni *sequences. The database includes more than 160,000 EST sequences from six developmental stages of *S. mansoni *– egg, miracidium, cercaria, germball (= sporocyst), schistosomulum, and mixed sex adults [30]. Significant hits were found to ESTs from all six of these stages (not shown). Representative accession numbers of the positive matches are presented in Additional files 6 and 7, along with brief details of the regions where the matches were located and statistical significance of the matches. In brief, positive ESTs spanning all of the 5'UTR, 3'UTR, EN and RT were located in most of these developmental stages. Based on these findings, it appeared that *SR3 *was expressed in developmental stages throughout the life cycle of *S. mansoni*.

### SR3 integration sites

**Figure 6:**
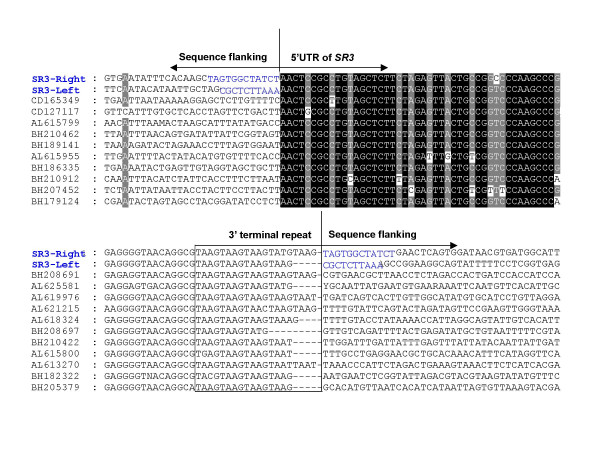
Multiple sequence alignment of the nucleotide sequences flanking the insertion sites of copies of the *SR3 *non-LTR retrotransposon within the genome of *Schistosoma mansoni*. Sequences flanking the 5'UTR of *SR3-left *and *SR3-right *are aligned in the top panel, while those flanking the 3'-terminus of *SR3-left *and *SR3-right *are presented in the bottom panel. Target site duplications are evident at the sites of *SR3-left *and *SR3-right *integration, and are indicated with bold font. Conservation of residues is indicated by the shading of boxes. Target sequences were identified among entries in the GSS database of *S. mansoni *sequences at GenBank or the Sanger Institute [39, 40].

In order to investigate the nature of integration sites or target sequences of the new retrotransposon within the schistosome genome, five kilobases of nucleotide sequences flanking the 5'- and 3'-UTRs of both *SR3-left *and *SR3-right *were employed as queries to search the GenBank non-redundant nucleotide and protein databases, and the GSS and EST entries for *S. mansoni*. These searches revealed no significant matches to any sequences encoding genes of *Schistosoma *species (not shown). However, they did reveal that *SR3 *elements appear to target AT-rich sites, as indicated in Figure 6, a similar preference to *L1 *retrotransposons within the human genome [31,32]. More specifically, the average AT content of the integration sites of the 21 copies of *SR3 *shown in Figure 6 was 68 % AT. Whereas target site specificity does not appear to be stringent for *SR3*, it can be expected to reflect the recognition sequence of the *SR3 *endonuclease. For example, L1 elements apparently integrate at numerous sites in the genome because the endonuclease of L1 preferably cleaves DNA at the short consensus sequence, 5'-TTTT/A-3', where/designates the cleavage site [31,33].

To propagate, non-LTR retrotransposons employ their EN and RT enzymes respectively to nick a genomic target site and reverse transcribe the retrotransposon, integrating the element into a new genomic locus [33-35]. This process is termed target-site-primed reverse transcription. For the L1 elements in the human genome, a new L1 insertion is usually flanked by short direct repeats derived from the target DNA locus upon L1 integration [32,36]. These repeats are called target site duplications (TSDs), and can range from several to several hundred nucleotides in length [32,37]. Interestingly, both *SR3-left *and *SR3-right *are flanked by TSDs of 10 and 12 bp, respectively; TAGTGGCTAATCT for *SR3-right *and CGCTCTTAAA for *SR3-left *(Fig. 6). The presence of these TSDs provides further indication, along with their intact structure, of recent activity of these two copies of *SR3 *localized in BAC 49_J_14 [see [32]]. Apparently unlike *SR3*, and certainly unlike L1, some other clades of non-LTR retrotransposons exhibit extreme target site specificity, the well-known examples being the *R2 *and *R4 *elements which are found exclusively in the ribosomal RNA genes of insects (e.g., *Bombyx mori*, *Drosophila melanogaster*) and nematodes (e.g., *Ascaris lumbricoides*) or in simple repeats (e.g., the *Dong *element from *B. mori*) [25].

Nonetheless, as noted above, we have detected the presence of *SR3 *of *S. haematobium *within introns of the AChE gene [28], and in addition, other RTE elements have been reported from gene-rich sites of the schistosome genome. The degenerate copy of a non-LTR retrotransposon, *SR2 *[18] in BAC clone BAC 49_J_14 has integrated into intron 1 of the Zn-Cu superoxide dismutase (SOD) gene of *S. mansoni *(Figure 1). *SR2 *from schistosomes has been recorded from several other target genes including 28 kDa glutathione *S *transferase [18], cathepsin D [20] and the UTR of heat shock protein 70 [10]. Furthermore, the *RTE-1 *retrotransposon of *C. elegans *was found inserted in the intron of *pim related kinase*-1 (*prk*-1) gene [27]. Thus, although *SR3 *and other RTE clade retrotransposons do not exhibit tight target site specificity, they seem to prefer to integrate into AT-rich sites and, in addition, are frequently found in introns and other-non coding areas of protein encoding gene loci.

## Conclusion

A new non-LTR retrotransposon, *SR3*, is reported from the genome of the human blood fluke *Schistosoma mansoni*. Numerous copies of *SR3 *are interspersed throughout the *S. mansoni *genome, and given the apparently intact sequence of the *SR3-right *copy of *SR3 *located in BAC 49_J_14 and the presence of transcripts from at least six developmental stages of *S. mansoni*, *SR3 *appears to be an active or recently active retrotransposon. This element is also present in the related human schistosomes, *S. haematobium *and *S. japonicum*. Based on phylogenetic comparisons of both the reverse transcriptase and endonuclease domains, SR3 represents a distinct family of RTE elements, discrete from the SR2 family described previously from schistosomes [18]. While there are numerous non-LTR retrotransposons in the schistosome genome, most elements so far described belong either to the RTE clade or CR1 clades [4], both of which are considered to be more advanced clades of non-LTR retrotransposons with progressive features including lack of target site specificity and an ORF encoding endonuclease and reverse transcriptase, respectively [25]. The presence of these and the apparent absence of some other clades of non-LTR retrotransposons should be informative in understanding the influence of mobile genetic elements in shaping the schistosome genome and its evolution and in studies of the phylogeny of schistosomes and related taxa. Finally, for studies with transgenesis of schistosomes, it may be possible to adapt an active copy of *SR3 *– such as *SR3-right *– for the introduction of transgenes into the schistosome genome in similar fashion to the adaptation of *L1 *elements of humans for studies on the movement of LINE elements in cultured human cell lines [23,37,38].

## Methods

### Bioinformatics approaches for detection of mobile sequences in the schistosome genome

The keyword phrase <Reverse Transcriptase> was used as the query to search the EST_others and GSS databases at GenBank for novel schistosome sequences associated with mobile genetic elements. Schistosome RT-like sequences that were retrieved were employed subsequently to search for matches in the GenBank non-redundant sequence database using BLASTn, BLASTx and/or tBLASTn [39]. Sequences of the previously characterized schistosome retrotransposons including *Gulliver, pido, SjR2 *of *S. japonicum *[4] and *Boudicca *[12] also were employed as queries. In addition, retrotransposon integration sites were investigated by interrogation of the *S. mansoni *genome survey sequences (GSS) at the Sanger Institute, Hinxton, U.K [40].

### Parasites and parasite DNA

The life cycle of *Schistosoma mansoni *(NMRI strain, of Puerto Rican origin) was maintained at the Queensland Institute of Medical Research, Brisbane, Australia using experimentally infected mice and albino *Biomphalaria glabrata *snails. Genomic DNAs (gDNAs) of adult mixed sex parasites perfused from mice and cercariae (shed from snails) of *S. mansoni *were extracted using Qiagen's Genomic Tip-100 system according to the manufacturer's instructions.

### Southern hybridization

Thirty micrograms of *S. mansoni *gDNA was cleaved with restriction enzymes *Hin*d III, *Eco*R I, *Bam*H I and *Xho *I. Digested gDNA was fractionated through 0.8% agarose gel and then was transferred to nylon membrane (Hybond-N+, Amersham Biosciences) by capillary action [41]. Southern hybridization analysis was performed using a horseradish peroxidase labelled probe and the ECL detection system (Amersham Biosciences). The membrane was incubated in hybridization medium (provided with kit) supplemented with the labeled probe overnight at 42°C, after which the membrane was washed in 0.4% SDS, 0.5× SSC at 42°C (two washes, 20 min. each) and subsequently in 2× SSC at room temperature (two washes, 5 min. each). The retrotransposon-like gene probe was amplified by polymerase chain reaction (PCR) with specific primers using *S. mansoni *gDNA as a template. Specific primers targeting the amplification of the RT domain of *SR3 *were *SR3-forward*, 5'-GAAGATTTGGGAAGAGGAACA and *SR3-reverse*, 5'-AACGATGCTCCCCAGATAAT (spanning nucleotides 1,809–2,622, Additional file 1). The *SR3-right *gene probe was amplified using specific primer SR3 forward (same as for the *SR3-left *probe) and SR3-right reverse 5'-CAACGATGCTCCCCAGGTACTTG (nt 1,809–2,622). Probes were sized in gels, isolated and purified before use. These probe sequences have been assigned GenBank accession numbers DQ008120 and DQ008121 for the *SR3-left*- and *SR3-right*-based probes, respectively.

### Sequence analysis and phylogenetic analysis of new retrotransposons

The amino acid sequences of the functional domains of both RT and EN of both copies of the new non-LTR retrotransposon were aligned to other non-LTR retrotransposons by the ClustalW method [42] using BioEdit software [43] and optimized gaps and errors were referenced to conserved domains defined by Malik et al. [25]. Edited sequence alignments of the RT and EN domains were analyzed for phylogenetic relationships using the PHYLIP package [44]. Phylograms were generated and assessed for bootstrap values of 1,000 replicates using the neighbor-joining method with assistance from SEQBOOT and NEIGHBOR in the PHYLIP software suite [44]. Trees were displayed by TreeView [45]. Sequences used in the phylogenetic analyses were obtained from the GenBank, EMBL and PIR databases. They included family representatives from 11 major clades of non-LTR retrotransposons [25]. RT sequences of Group II introns from bacteria and EN sequences from bacteria were used as outgroups for the RT and EN trees, respectively. The names and accession numbers of the aligned sequences were: *SR1 *(U66331), *SR2 *(AF025672), *Perere *(BK004067) and *Perere *3 (BN000794) from *S. mansoni*, *SjR2 *(AY027869) and *pido *(AY034003) from *S. japonicum*, *ShR3 *(AY167025) from *S. haematobium*, *RTE1 *from *C. elegans *(AF054983), *JAM1 *(Z86117) and *Lian *(U87543) from *Ae. aegypti*, Bov-B LINE from *Vipera ammodytes *(AF332697), *Branchiostoma floridae *clone CH302-99K22 (AC150430), BDDH from *Bos taurus *(AC150753), BCNT from *Tragulus javanicus *(AB191483), ENSANGP00000028171 from *Anopheles gambiae *strain PEST (XM556470), *Tx1 *from *Xenopus laevis *(M26915), *Swimmer *from the medaka fish, *Oryzias latipes *(AF055640), *L1 *from the rat (U83119), *L1 *from the mouse (AF081114), *L1 *from the human (U93574), *R4 *from *Ascaris lumbricoides *(U29445), *R2 *from *Bombyx mori *(M16558), *R2 *from the earwig, *Forficular auricularia *(AF015819), *R2 *from *Drosophila melanogaster *(X51967), *CZAR *from *Trypanosoma cruzi *(M62862), *CRE2 *from *Crithidia fasciculata *(U19151), *CRE1 *from *C. fasciculata *(M33009), *CR1 *from the turtle *Platymys spixii *(AB005891), *CR1 *from the chicken (U88211), *Q *from *Anopheles gambiae *(U03849), *Tad1 *from *Neurospora crassa *(L25662), *CgT1 *from the fungal phytopathogen, *Colletotrichum gloeosporioides *(L76169), *R1 *from *B. mori *(M19755), *R1 *from *D. melanogaster *(X51968), *Tart *from *D. melanogaster *(U14101), *Juan *from *Ae. aegypti *(M95171), *Jockey *(M22874), *Doc *(X17551), and *I *(M14954) from *D. melanogaster*, Group II intron-encoding maturase from *Symbiobacterium thermophilum *(BAD41717), Group II intron protein from *Streptococcus pneumoniae *(CAI33690), AP1 endonuclease from *Paracentrotus lividus *(AAY37515), AP endonuclease from *Pseudomonas syringae *(AAY37515), and exonuclease III from *Escherichia coli *(NP288182).

### Copy number estimation

Estimates of the copy number of the *SR3 *retrotransposon were established by a comparative bioinformatics approach [12-14] wherein BLAST analysis of the BAC-end database of *S. mansoni *genomic sequences targeted more well-characterized retrotransposable elements from *S. mansoni*, and some other reference genes, for which copy numbers have been reported. These included the *Boudicca *and *Sinbad *LTR retrotransposons [12,13], the non-LTR retrotransposons *SR1 *and *SR2 *[18,22], the 18S ribosomal RNA genes, a middle repetitive element [46], and cathepsin D, a single copy gene [20]. The NCBI database was searched by BLAST using the sequences of these mobile genetic elements and some other genes of *S. mansoni*, all of which included at least one *Hin*d III site. Specifically, the Advanced BLAST function was used, set to search only the *S. mansoni *sequences in the GSS database (Limit by Entrez Query: <Schistosoma mansoni [organism]>), and with the E (Expect) value at 0.000001. This stringent cutoff value was used to minimize the chance of counting other *RTE-1*-like elements in the total copy number of *SR3*. Since the formula for E is based not only on the bit scores of the local alignment of each pair of sequences, but also on the lengths of the subject and query [47], no additional correction was made for the length of the query sequence. Only hits with a Blast score of ≥100 were counted.

### Investigation of integration sites

Five kilobases of the sequence flanking both 5'- and 3'-termini of *SR3-left *and *SR3-right *were employed as queries in BLAST searches of the non-redundant and dbEST GenBank databases limited by the organism [Schistosoma mansoni]. Sequences flanking additional copies of *SR3 *identified in other GenBank entries were also used as queries in BLAST searches to investigate the target site of *SR3 *integration. Multiple sequence alignments of integration sites were assembled and examined for target site preferences.

## List of abbreviations

LTR, long terminal repeat; RT, reverse transcriptase; EN, endonuclease; UTR, untranslated region; *SR3*, schistosome retrotransposon 3; ORF, open reading frame; BAC, bacterial artificial chromosome; AChE, acetylcholineesterase; GSS, genome survey sequence; TSD, target site duplication.

## Authors' contributions

TL carried out the sequence analysis, multiple sequence alignments, phylogenetic trees and drafted the manuscript. NK performed the Southern hybridization and assisted with the bioinformatics analyses. AL contributed to the experimental designs, sequence alignments, bioinformatics, and with drafting the manuscript. PJB oversaw the project, carried out copy number and other bioinformatics analyses, and drafted the manuscript. All authors read and approved the final manuscript.

## Supplementary Material

Additional File 1 Nucleotide and deduced amino acid sequence of the *SR3-left *retrotransposon. Click here for file

Additional File 2 Nucleotide and deduced amino acid sequence of the *SR3-right *retrotransposon. Click here for file

Additional File 3 Sequence alignment of deduced open reading frames of *SR3-left *and *SR3-right*. Click here for file

Additional File 4 Multiple sequence alignment of the reverse transcriptase domain of *SR3 *and related non-LTR retrotransposons. Click here for file

Additional File 5 Multiple sequence alignment of the endonuclease domain of *SR3 *and related non-LTR retrotransposons. Click here for file

Additional File 6 Table of representative GenBank accessions to show the presence in the *Schistosoma mansoni *transcriptomes of messenger RNAs encoding *SR3 *expressed in six developmental stages of the parasite. Click here for file

Additional File 7 Table of representative GenBank accessions to show the presence in the *Schistosoma mansoni *transcriptomes of messenger RNAs encoding *SR3 *expressed in six developmental stages of the parasite. Click here for file
