# Supplementary material for: Characterization of SR3 reveals abundance of non-LTR retrotransposons of the RTE clade in the genome of the human blood fluke, Schistosoma mansoni
Source: BMC Genomics. 2005 Nov 4;6:154. doi: 10.1186/1471-2164-6-154 (PMC1291365; doi:10.1186/1471-2164-6-154)
Supplement: Additional File 1 — Nucleotide and deduced amino acid sequence of the SR3-left retrotransposon. [file 1471-2164-6-154-S1.doc]

AACTCCGCCTGTAGCTCTTCTAGAGTTACTGCCGGTCCCAAGCCCGGGTAAAGGAGGAGGGTTAGGCATGGGGTTAGCGACCCCATCCCGTAGAAAAGCTAACTCGCTAAAAAAACGCTA 120

ACCAGAAAAAATAATTCAAACCATTTAAACTCTGCCCTCTCAGTTGAAGGAATAATTATGACGTCTCATGATGAAAGCCGGAATTCTTCGGAAGTCACGAGACCGATGCACCTTCTAACA 240
 M T S H D E S R N S S E V T R P M H L L T

**ENDO**

**I**

# II

ACCAGAGCAACACTCTTTATAGGTACATGGAACGTCCGGACAATGTGGGAGACAGGAAAGACCAGCCAAATAGCAATGGAAATGAGGAGATACAACTTGGCAGTACTCGGAATCAGCGAA 360
 T R A T L F I G T W N V R T M W E T G K T S Q I A M E M R R Y N L A V L G I S E

**II**

**III**

ACCCATTGGACACAAACTGGACAACAAAGGCTAGGTACAGGAGAGATGCTGCTGTACTCCGGTCACGAAGGGGAAAATGCTCCACACACTCAGGGAGTTGCTCTAATGCTGTCCAAAGAA 480
 T H W T Q T G Q Q R L G T G E M L L Y S G H E G E N A P H T Q G V A L M L S K E

**V**

GCACGAAATGCACTTGTGGGATGGGAATCTCATGGACCCAGGATAATCAAAGCATCATTCAGAACAAAGAAGCAAGGGATCACAATGAACGTTATCCAATGTTATGTACCCACCAATGAT 600
 A R N A L V G W E S H G P R I I K A S F R T K K Q G I T M N V I Q C Y V P T N D

**VI**

AGCAACGACGATGATAAAGATCAGTTCTATGAAAGGCTGCAATCAATTATAGAGAAGTGCTCACGAAAGGACCTCACCGTCCTGATGGGAGATCTGAATGCTAAAGTTGGAGTGGACAAC 720
 S N D D D K D Q F Y E R L Q S I I E K C S R K D L T V L M G D L N A K V G V D N

ACAGGATATGAAGATGTAATTGGACGACATGGATTAGGAGAGAGAAATGAAAATGGGGAGAGACTTGCAAACCTATGTGCATTCAACAAATTGGTTATAGGCGGCACAATATTCCCACAC 840
 T G Y E D V I G R H G L G E R N E N G E R L A N L C A F N K L V I G G T I F P H

**VIII**

AAGCGCATACACAAAGCTACATGGATCTCACCGGACCAAACCACAGAGAACCAGATAGATCACATCTGTATCAACAAAAAATTCCGAAGATCAATGGAAGATGTGAGAACCCGGAGAGGA 960
 K R I H K A T W I S P D Q T T E N Q I D H I C I N K K F R R S M E D V R T R R G

**IX**

GCTGACATAGCTTCAGATCACCACCTGGTTGTGGCCAAGATGAGACTGAAGCTAAAGAAACACTGGACAACTGCACAAACAGCACTACAAAGGCTCAATACAGCCTTCCTTCGAGATATT 1080
 A D I A S D H H L V V A K M R L K L K K H W T T A Q T A L Q R L N T A F L R D I

GACAAGCTCAATAAATTCAAGATAACTCTCAACAACAGGTTCCAAGCTCTACAGGATCTACTGAATGAACAAGAAACTACTTTGGAGGACAACTGGAAAGGGATAAAAGAAGTCCTAACT 1200
 D K L N K F K I T L N N R F Q A L Q D L L N E Q E T T L E D N W K G I K E V L T

TCAACGTGTCAGGAGGTTCTTGGTCCTAAGAAGCATCATCACAAGGAATGGATCTCTATGGGAACCCTGGACAAAATTCAAGAAAGGAAGAACAAGAGACTAGCAATTAACAAGAGCCGA 1320
 S T C Q E V L G P K K H H H K E W I S M G T L D K I Q E R K N K R L A I N K S R

ACACGAGCAGAGAAAGTCAAAGCACAAGCAGACTACGCAGAAGCAAACAGGGAAGTGAAGAAAAGCATTAAAGCCGACAAGCAGAAGTACATGGGAGAACTAGCAACGGCGGCGGAAAAA 1440
 T R A E K V K A Q A D Y A E A N R E V K K S I K A D K Q K Y M G E L A T A A E K

GCTACAAGAGAAGGGAATATGAAACAACTATATGATACAACGAAGAAATTGACAGGGAGATATAGCAAACCAGAGAGACCAGTCAAGGACAAAGAAGGAAAGACAATCACTGAGACTCAA 1560
 A T R E G N M K Q L Y D T T K K L T G R Y S K P E R P V K D K E G K T I T E T Q

GAACAGAGGAAAAGATGGGCAGAATACTTCGAGGAACTGCTGAATAGACCAGCCCCATTGAATCCACCGAACATCGAAGCAGCCCACACTGACCTTCCAATAGATGTCACTCCACCAACG 1680
 E Q R K R W A E Y F E E L L N R P A P L N P P N I E A A H T D L P I D V T P P T

ATCGAAGAAGTCAAGATGGCCGTCAGACAAATCAAAAATGGGAAGGCGGCAGGACCTGACAATATACCAGCAGAAGCACTGAAGTCAGACATTGAAATAACTGCAAACATGCTTCACCTT 1800
 I E E V K M A V R Q I K N G K A A G P D N I P A E A L K S D I E I T A N M L H L

**1**

**RT**

CTATTCAAGAAGATTTGGGAAGAGGAACAAGTGCCAATGGACTGGAAAGAAGGATATCTCATCAAGATACCAAAGAAAGGAGATCTGAGCAAATGTGAGAACTACAGAGGCATCAGTTTG 1920
 L F K K I W E E E Q V P M D W K E G Y L I K I P K K G D L S K C E N Y R G I S L

**2**

TTATCAGTACCAGGAAAAGTCTTCAACAGAGTGCTGCTGAACCTGATGAAAGACGCAGTAGACGCCGAACTTAGGGATCAACAGGCTGGATTCTGTAAGGATAGATCGTGCACAGACCAG 2040
 L S V P G K V F N R V L L N L M K D A V D A E L R D Q Q A G F C K D R S C T D Q

**3**

ATTGCGACACTACGGATCATCGTAGAACAATCAGTTGAGTGGAACTCATCACTACACGTCAACTTCATCGACTATGAGAAGGCGTTTGACAGCGTGGATAGGAGAACATTATGGAATCTT 2160
 I A T L R I I V E Q S V E W N S S L H V N F I D Y E K A F D S V D R R T L W N L

**3**

CTTCGACACTATGGAGTTCCTGAAAAAATTGTCAACATTATCCAAAACTCATACGATGGACTACAGTGCAAAGTGGTGCATGGAGGACAGCTGACAGATGCATTTCCAGTAAGGACCGGA 2280
 L R H Y G V P E K I V N I I Q N S Y D G L Q C K V V H G G Q L T D A F P V R T G

**4**

GTCAGACAAAGCTGTCTACTCTCCCCATTCCTCTTCCTTCTAGTGATTGACTAGATTATGAAGAATTCGACATCTGACGGGAAATACGGAATACAATGGACAGCTCAGAATCAATTAGAT 2400
 V R Q S C L L S P F L F L L V I D * I M K N S T S D G K Y G I Q W T A Q N Q L D

**6**

**5**

GATTTGGACTTCGCAGATGACCTAGCCCTTCTCTCTCATACACACGAACAAATGCAGATGAAGACAGCAGATGTAGCAGCAGCCTCCGCATCGATAGGCCTCCACATTCACAAAGGAAAA 2520
 D L D F A D D L A L L S H T H E Q M Q M K T A D V A A A S A S I G L H I H K G K

**7**

**RT**

AGCAAGATTCTCAAATGCAATACGGAGAACACCAATCCAATCACACTTGATGGCGAAACCCTGGAAGAGGTGGAAACATTCAATTATCTGGGGAGCATCGTTGATAAACAAGGAGGATCT 2640
 S K I L K C N T E N T N P I T L D G E T L E E V E T F N Y L G S I V D K Q G G S

GATGCAGATGTAAAGGCGAGGATTGGCAAAGCAAGGGAAGCATTTCGACAATTGAAGAACATATGGAACTCAAAACAATTCTCAACCAATTTCAAGGTCAGAATCTTTAATACGAACGTC 2760
 D A D V K A R I G K A R E A F R Q L K N I W N S K Q F S T N F K V R I F N T N V
AAGACAGTCCTACTGTATGGAGCTGAAACGTGGAGAACTACTACGACCATCATCAGGAAGGTACAAGTATTTATAAATAGTTGTCTACGCAAAATACTCAACATTCATTGGCCGGATACT 2880
 K T V L L Y G A E T W R T T T T I I R K V Q V F I N S C L R K I L N I H W P D T
ATCAGCAACAGTCTTCTGTGGGAGAGGACAAACTAGCTTCCAGCTGAAGGGAAAATTAGGAAAAGACGTTGGAACTGGATCGGACATACATTAAGGAAATCACCAATGTGCATTACAAGG 3000
 I S N S L L W E R T N * L P A E G K I R K R R W N W I G H T L R K S P M C I T R
CAATCCCTAACTTAGAATCCGGAAAGGAAGCGAAAAAACGGAAGGCCAAAAAACACATTACTCCGAGAAATAGAAGCAGATATGAAAAGGATGAATGTTAACTGGAAAGAATTGGAAAGG 3120
 Q S L T *
AAGGCTCAGGACAGAGTTGGATGAAGAATTCGAATGAGCGGCCTATGCTCTTCGACGAGGGGTAACAGGCG**TAAGTAAGTAAGTAAG** 3207
