# Supplementary material for: Characterization of SR3 reveals abundance of non-LTR retrotransposons of the RTE clade in the genome of the human blood fluke, Schistosoma mansoni
Source: BMC Genomics. 2005 Nov 4;6:154. doi: 10.1186/1471-2164-6-154 (PMC1291365; doi:10.1186/1471-2164-6-154)
Supplement: Additional File 2 — Nucleotide and deduced amino acid sequence of the SR3-right retrotransposon. [file 1471-2164-6-154-S2.doc]

AACTCCGCCTGTAGCTCTTCTAGAGTTACTGCCGGCCCCAAGCCCGGGTAAAGGAGGAGGATTGGGCATGGGGTTAACGACCCCATCCCGTAGAAAAGCTAACTCGCTAAAAAAACGCTA 120

TCCAGAAAAAATAATTCAAACCATTTAAACTCTGCCCTGAGAGTTGAAGGAATGATTATGACGTCTCATGATGAAAGCCGAAATTCTTCGGAAGTCACGAGACCGATGCACCTTCTAACA 240
 M T S H D E S R N S S E V T R P M H L L T

**ENDO**

**I**

**IX**

**RT**

**RT**

**II**

**II**

**III**

**VIII**

**VI**

**V**

**ENDO**

**7**

**6**

**5**

**4**

**3**

**3**

**2**

**1**

ACCAGAGCAACACTCTTTATATGTACATGGAACGTCCGGACAATGTGGGAGACAGGAAAGAGCAGCCAAATAGCAATGGAAATGAGGAGATACAACTTGGCAGTACTCGGAATCAGCGAA 360
 T R A T L F I C T W N V R T M W E T G K S S Q I A M E M R R Y N L A V L G I S E

ACCCATTGGACACAAACTGGACAACAAAGGCTAGGTACAGGAGAGATGCTGCTGTACTCCGGTCACGAAGGGGAAAATGCTCCACACATTCAGGGAGTTGCTCTAATGCTGTCCAAAGAA 480
 T H W T Q T G Q Q R L G T G E M L L Y S G H E G E N A P H I Q G V A L M L S K E

GCACGAAATGCACTTGTGGGATGGGAATCTCATGGACCCAGGATAATCAAAGCATCATTCAGAACAAAGAAGGAAGGGATCACAATGAACGTTATCCAATGTTATGCACCCACCAATGAT 600
 A R N A L V G W E S H G P R I I K A S F R T K K E G I T M N V I Q C Y A P T N D

AGCAACGACGATGATAAAGATCAGTTCTATGAAAGGCTTCAATCAATTATAACGCAGTGCTCACGAAAGGACCTCACCATCCTGATGGGGGATCTAAATGCTAAAGTTGGAGTGGACAAC 720
 S N D D D K D Q F Y E R L Q S I I T Q C S R K D L T I L M G D L N A K V G V D N

ACAGGATATGAAGATGTAATTGGACGACATGGATTAGGAGAGAGAAATGAAAATGGGGAGAGACTTGCAAACCTATGTGCATTCAACAAATTGGTTATAGGCGGCACAATATTCCCACAC 840
 T G Y E D V I G R H G L G E R N E N G E R L A N L C A F N K L V I G G T I F P H

AAGCGCATACACAAAGCTACATGGATCTCACCGGACCAAACCACAGAGAACCAGATAGATCACATCTGTATCAACAAAAAATTCCGAAGATCAATGGAAGATGTGAGAACCCGGAGAGGA 960
 K R I H K A T W I S P D Q T T E N Q I D H I C I N K K F R R S M E D V R T R R G

GCTGACATAGCTTCAGATCACCATCTGGTTGTGGCCAAGATGAGACTGAAGCTAAAGAAACACTGGACAACTGGACAAACAGCACTACAAAGGTTCAATACAGCCTTCCTTCGAGATACT 1080
 A D I A S D H H L V V A K M R L K L K K H W T T G Q T A L Q R F N T A F L R D T

GACAAGCTCCATGAATTCAAGATAACTCTCAACAACAGGTTCCAGGCTCTACAGGATCTACTGAAAGAACAAGAAACTACTTTGGAGGACAACTGGAAAGGGATAAAAGAAGTCCTAACT 1200
 D K L H E F K I T L N N R F Q A L Q D L L K E Q E T T L E D N W K G I K E V L T

TCAACGTGCCAGGAGGTTCTTGGTCCTAAGAAGCATCATCACAAGGAATGGATCTCTATGGGAACCCTGGACAAAATTCTAGAAAGGAAGAACAAGAAACTAGCAATTAACAACAGCCGA 1320
 S T C Q E V L G P K K H H H K E W I S M G T L D K I L E R K N K K L A I N N S R

ACACGAGCAGAGAAAGTCAAAGCACAAGCAGACTACGCAGAAGCAAACAGGGAAGTGAAGCAAAGCATTAAAGCCGACAAGCAGAAATACATGGGAGAACTAGCAACGACGGCGGAAAAA 1440
 T R A E K V K A Q A D Y A E A N R E V K Q S I K A D K Q K Y M G E L A T T A E K

GCTGCAAGAGAAGGGAATATGAAACAACCATATGATACAACGAAGAAATTGGCAGGGAGATATAGCAAACCAGAGAGACCAGTCAAGGACAAAGAAGGAAAGACAATCACTGAGATTCAA 1560
 A A R E G N M K Q P Y D T T K K L A G R Y S K P E R P V K D K E G K T I T E I Q

GAACAGAGGAAAAGATGGGCAGAATACTTCGAGGAACTGCTGAATAGACCAGCCCCATTGAATCCACCGAACATCGAAGCAGCCCACACTGACCTTCCAATAGATGTCACTCCACCAACG 1680
 E Q R K R W A E Y F E E L L N R P A P L N P P N I E A A H T D L P I D V T P P T

ATCGAAGAAGTGAAGATGGCCATCAGACAAATCAAAAGTGGGGAGGCGGCAGGACCTGACAATATACCAGCAGAAGCACTGAAGTCAGACATTGAAATAACTGCAAATATGCTTCACCTT 1800
 I E E V K M A I R Q I K S G E A A G P D N I P A E A L K S D I E I T A N M L H L

CTATTCAA**GAAGATTTGGGAAGAGGAACAA**GTGCCGATGGACTGGAAAGAAGGATATCTCATCAAGATACCAAAGAAAGGAGATCTGAGCAAATGTGAAAACTACAGAGGCATCAGTTTG 1920
 L F K K I W E E E Q V P M D W K E G Y L I K I P K K G D L S K C E N Y R G I S L

TTATCAGTACCAGGAAAAGTTTTCAACAGAGTGCTGCTGAATCGGATGAAAGACACAATAGACGCCGAACTTAGGGATCAACAGGCTGGATTCCGTAGGGATAGGTCATGCACAGACCAG 2040
 L S V P G K V F N R V L L N R M K D T I D A E L R D Q Q A G F R R D R S C T D Q

ATTGCGACACTACGGATCATCGTTGAACAATCAGTTGAGTGGAACTCATCACTACACGTCAACTTCATTGACTATGAGAAGGCGTTTGACAGCGTGGACAGGAGAACATTATGGAAACTT 2160
 I A T L R I I V E Q S V E W N S S L H V N F I D Y E K A F D S V D R R T L W K L

CTTCGACACTATGGAGTTCCTGAAAAGATTGTCAACATTATCCGGGACTCATACGATGGACTACAGTGCAAAGTGGTGCATGGAGGACAGCTGACAGATGCATTTCCAGTAAGGACCGGA 2280
 L R H Y G V P E K I V N I I R D S Y D G L Q C K V V H G G Q L T D A F P V R T G

GTCAGACAAGGCTGTCTACTCTCCCCATTCCTCTTCCTTCTGGTGATTGACTGGATTATGAAGGATTCGACATCTGACGGGAAATACGGAATACAATGGACAGCTCAGAATCAATTAGAT 2400
 V R Q G C L L S P F L F L L V I D W I M K D S T S D G K Y G I Q W T A Q N Q L D

GATTTGGACTTCGCAGATGACCTAGCCCTCCTCTCTCATACACACGAACAAATGCAGATGAAGACCGCAAATGTAGCAGCAGCCTCCGCATCGATAGGCCTCCACATTCACAAAGGAAAA 2520
 D L D F A D D L A L L S H T H E Q M Q M K T A N V A A A S A S I G L H I H K G K

AGCAAGATTCTCAAATTCAACACGGAGAACACCAACCTAATCACACTTGATGGTGAAACTCTGGAAGAGGTGGAAACATT**CAAGTACCTGGGGAGCATCGTT**GATAAACAAGGAGGATCG 2640
 S K I L K F N T E N T N L I T L D G E T L E E V E T F K Y L G S I V D K Q G G S

GATGCAGATGTAAAGGCGAGGATTGGCAAAGCAAGGGCAGCATTTTTACAAATGAAGAACATATGGAACTCAAAACAACTCTCAACCAATTTCAAGGTCAGAATATTTAATACGAACGTA 2760
 D A D V K A R I G K A R A A F L Q M K N I W N S K Q L S T N F K V R I F N T N V

AAGACAGTCCTACTGTATGGAGCTGAAACGTGGAGAACTACTACGACCATCATCAGGAAGGTACAAGTATTTATAAACAGTTGTTTACGCAAAATACTCAACATTCATTGGCCGGATGCT 2880
 K T V L L Y G A E T W R T T T T I I R K V Q V F I N S C L R K I L N I H W P D A

ATCAGCAACAGCGTTTTATGGGAGAGGAGAAACCAGCTTCCAGCTGAAGAGGAAATTAGGAAATGACGTTGGAAGTGGATCGGACATACATTAAGCAAATCACCAATGTGCATCGCGACT 3000
 I S N S V L W E R R N Q L P A E E E I R K *

CAATCCCTAACTTGGAATCCGGAATGGAAGCGGAAAAGAGGAAGGCCAAAGAACACACTACGCCGGGAAATAGAAGCCGATATGAAAAGGCTGAATAGCAACTGGAAAGAACTGGAAAGG 3120

AAGGCTCAGGACAGAGTTGGATGGAGAATGCTGGTGAGCGGCCTATGCTCCTCGACGAGGGGTAACAGGCG**TAAGTAAGTAAGTATGTAAG** 3211
