# Supplementary material for: Characterization of SR3 reveals abundance of non-LTR retrotransposons of the RTE clade in the genome of the human blood fluke, Schistosoma mansoni
Source: BMC Genomics. 2005 Nov 4;6:154. doi: 10.1186/1471-2164-6-154 (PMC1291365; doi:10.1186/1471-2164-6-154)
Supplement: Additional File 5 — Multiple sequence alignment of the endonuclease domain of SR3 and related non-LTR retrotransposons. [file 1471-2164-6-154-S5.doc]

**SR3-right**

**SR3-left**

**SR3-left**

**SR3-right**

**SR3-left**

**SR3-right**

**III**

**II**

**I**

**V**

**VI**

**IX**

**VIII**
