# Supplementary material for: Characterization of SR3 reveals abundance of non-LTR retrotransposons of the RTE clade in the genome of the human blood fluke, Schistosoma mansoni
Source: BMC Genomics. 2005 Nov 4;6:154. doi: 10.1186/1471-2164-6-154 (PMC1291365; doi:10.1186/1471-2164-6-154)
Supplement: Supplementary file 5 [file 1471-2164-6-154-S3.doc]

```
SR3-Left  : MTSHDESRNSSEVTRPMHLLTTRATLFIGTWNVRTMWETGKTSQIAMEMRRYNLAVLGISETHWTQTGQQRLGTGEMLLY :  80
SR3-Right : MTSHDESRNSSEVTRPMHLLTTRATLFICTWNVRTMWETGKSSQIAMEMRRYNLAVLGISETHWTQTGQQRLGTGEMLLY :  80

SR3-Left  : SGHEGENAPHTQGVALMLSKEARNALVGWESHGPRIIKASFRTKKQGITMNVIQCYVPTNDSNDDDKDQFYERLQSIIEK : 160
SR3-Right : SGHEGENAPHIQGVALMLSKEARNALVGWESHGPRIIKASFRTKKEGITMNVIQCYAPTNDSNDDDKDQFYERLQSIITQ : 160

SR3-Left  : CSRKDLTVLMGDLNAKVGVDNTGYEDVIGRHGLGERNENGERLANLCAFNKLVIGGTIFPHKRIHKATWISPDQTTENQI : 240
SR3-Right : CSRKDLTILMGDLNAKVGVDNTGYEDVIGRHGLGERNENGERLANLCAFNKLVIGGTIFPHKRIHKATWISPDQTTENQI : 240

SR3-Left  : DHICINKKFRRSMEDVRTRRGADIASDHHLVVAKMRLKLKKHWTTAQTALQRLNTAFLRDIDKLNKFKITLNNRFQALQD : 320
SR3-Right : DHICINKKFRRSMEDVRTRRGADIASDHHLVVAKMRLKLKKHWTTGQTALQRFNTAFLRDTDKLHEFKITLNNRFQALQD : 320

SR3-Left  : LLNEQETTLEDNWKGIKEVLTSTCQEVLGPKKHHHKEWISMGTLDKIQERKNKRLAINKSRTRAEKVKAQADYAEANREV : 400
SR3-Right : LLKEQETTLEDNWKGIKEVLTSTCQEVLGPKKHHHKEWISMGTLDKILERKNKKLAINNSRTRAEKVKAQADYAEANREV : 400

SR3-Left  : KKSIKADKQKYMGELATAAEKATREGNMKQLYDTTKKLTGRYSKPERPVKDKEGKTITETQEQRKRWAEYFEELLNRPAP : 480
SR3-Right : KQSIKADKQKYMGELATTAEKAAREGNMKQPYDTTKKLAGRYSKPERPVKDKEGKTITEIQEQRKRWAEYFEELLNRPAP : 480

SR3-Left  : LNPPNIEAAHTDLPIDVTPPTIEEVKMAVRQIKNGKAAGPDNIPAEALKSDIEITANMLHLLFKKIWEEEQVPMDWKEGY : 560
SR3-Right : LNPPNIEAAHTDLPIDVTPPTIEEVKMAIRQIKSGEAAGPDNIPAEALKSDIEITANMLHLLFKKIWEEEQVPMDWKEGY : 560

SR3-Left  : LIKIPKKGDLSKCENYRGISLLSVPGKVFNRVLLNLMKDAVDAELRDQQAGFCKDRSCTDQIATLRIIVEQSVEWNSSLH : 640
SR3-Right : LIKIPKKGDLSKCENYRGISLLSVPGKVFNRVLLNRMKDTIDAELRDQQAGFRRDRSCTDQIATLRIIVEQSVEWNSSLH : 640

SR3-Left  : VNFIDYEKAFDSVDRRTLWNLLRHYGVPEKIVNIIQNSYDGLQCKVVHGGQLTDAFPVRTGVRQSCLLSPFLFLLVID*I : 719
SR3-Right : VNFIDYEKAFDSVDRRTLWKLLRHYGVPEKIVNIIRDSYDGLQCKVVHGGQLTDAFPVRTGVRQGCLLSPFLFLLVIDWI : 720

SR3-Left  : MKNSTSDGKYGIQWTAQNQLDDLDFADDLALLSHTHEQMQMKTADVAAASASIGLHIHKGKSKILKCNTENTNPITLDGE : 799
SR3-Right : MKDSTSDGKYGIQWTAQNQLDDLDFADDLALLSHTHEQMQMKTANVAAASASIGLHIHKGKSKILKFNTENTNLITLDGE : 800

SR3-Left  : TLEEVETFNYLGSIVDKQGGSDADVKARIGKAREAFRQLKNIWNSKQFSTNFKVRIFNTNVKTVLLYGAETWRTTTTIIR : 879
SR3-Right : TLEEVETFKYLGSIVDKQGGSDADVKARIGKARAAFLQMKNIWNSKQLSTNFKVRIFNTNVKTVLLYGAETWRTTTTIIR : 880

SR3-Left  : KVQVFINSCLRKILNIHWPDTISNSLLWERTN*LPAEGKIRK : 920
SR3-Right : KVQVFINSCLRKILNIHWPDAISNSVLWERRNQLPAEEEIRK : 922
```
